# Saccharification of newspaper waste after ammonia fiber expansion or extractive ammonia

**DOI:** 10.1186/s13568-016-0189-9

**Published:** 2016-03-02

**Authors:** Salvatore Montella, Venkatesh Balan, Leonardo da Costa Sousa, Christa Gunawan, Simona Giacobbe, Olimpia Pepe, Vincenza Faraco

**Affiliations:** Department of Chemical Sciences, University of Naples ‘‘Federico II’’, Complesso Universitario Monte S. Angelo, Via Cintia, 4, 80126 Naples, Italy; Department of Chemical Engineering and Materials Science, DOE Great Lakes Bioenergy Research Center, Michigan State University, East Lansing, MI 48823 USA; Department of Agriculture, University of Naples “Federico II”, Portici, Naples, Italy

**Keywords:** AFEX pretreatment, Arabinofuranosidase, Biorefining, Cellulase, EA pretreatment, Hemicellulase, Municipal solid waste, Newspaper waste

## Abstract

The lignocellulosic fractions of municipal solid waste (MSW) can be used as renewable resources due to the widespread availability, predictable and low pricing and suitability for most conversion technologies. In particular, after the typical paper recycling loop, the newspaper waste (NW) could be further valorized as feedstock in biorefinering industry since it still contains up to 70 % polysaccharides. In this study, two different physicochemical methods—ammonia fiber expansion (AFEX) and extractive ammonia (EA) were tested for the pretraetment of NW. Furthermore, based on the previously demonstrated ability of the recombinant enzymes endocellulase rCelStrep, α-l-arabinofuranosidase rPoAbf and its evolved variant rPoAbf F435Y/Y446F to improve the saccharification of different lignocellulosic pretreated biomasses (such as corn stover and *Arundo donax*), in this study these enzymes were tested for the hydrolysis of pretreated NW, with the aim of valorizing the lignocellulosic fractions of the MSW. In particular, a mixture of purified enzymes containing cellulases, xylanases and accessory hemicellulases, was chosen as reference mix and rCelStrep and rPoAbf or its variant were replaced to EGI and Larb. The results showed that these enzymatic mixes are not suitable for the hydrolysis of NW after AFEX or EA pretreatment. On the other hand, when the enzymes rCelStrep, rPoAbf and rPoAbf F435Y/Y446F were tested for their effect in hydrolysis of pretreated NW by addition to a commercial enzyme mixture, it was shown that the total polysaccharides conversion yield reached 37.32 % for AFEX pretreated NW by adding rPoAbf to the mix whilst the maximum sugars conversion yield for EA pretreated NW was achieved 40.80 % by adding rCelStrep. The maximum glucan conversion yield obtained (45.61 % for EA pretreated NW by adding rCelStrep to the commercial mix) is higher than or comparable to those reported in recent manuscripts adopting hydrolysis conditions similar to those used in this study.

## Introduction

The world municipal solid waste (MSW) volume is expected to reach 2.2 billion tonnes by 2025, due to the increase of urban residents to 4.3 billion. Improperly managed urban solid waste is one of the main causes of environmental pollution and a serious health hazard, due to contamination of groundwater and surface water by leachate, as well as air pollution by their burning. Monitoring of pollution from different waste management options is crucial and environmental concerns are increasing the focus on the reuse and the recycling of MSW and on processes for adding value to wastes. In particular, the lignocellulosic fractions of MSW can be a source of a wide range of high added value products by biotechnological “tailor made” processes (Liguori et al. 2013) for the biorefineries development (Menon and Rao 2012; Fava et al. 2015; Esposito and Antonietti 2015). The future of biorefining industry depends mostly on the availability of cheap, sustainable and abundant biomasses as feedstock. The lignocellulosic fractions from MSW are more suitable to achieve this objective than dedicated agricultural crops (avoiding the displacement of food crops and minimizing the conflict food versus fuel) or forest resources (avoiding the issues concerning a massive deforestation). Moreover the change of land use and the removal of forest trees in large areas can have a negative impact on the ecosystem. The lignocellulosic fractions from MSW show further advantages such as widespread availability, predictable and low pricing, and suitability for most conversion technologies.

Among the lignocellulosic fractions of MSW, paper waste represents approximately 30 % of MSW and it is the second most abundant lignocellulose fraction after humid fraction (U.S. Environmental Protection Agency—EPA 2015). The major method of paper waste management is recycling: according to EPA, the paper waste can typically be recycled from 5 to 7 times, before papermaking fibers become too short and weak to hold together; each recycling requires de-inking with chemicals processing and by adding virgin wood fibers. The newspaper waste (NW) alone constitutes up to 14 % of MSW (Subhedar et al. 2015), and it can be recycled fewer times that office paper due to the fact that it is usually made by shorter fibers. After the typical paper recycling loop, the NW can be further valorized as feedstock in biorefinering industry since it still contains up to 70 % polysaccharides which can be hydrolysed to fermentable sugars (Wang et al. 2012a).

For an effective saccharification, the NW, as all the lignocellulose biomasses, requires a physical, chemical or enzymatic pretreatment to break down the recalcitrant lignin and increase the polysaccharides accessibility for the following hydrolysis. The latter one can be performed by enzymatic method, that is more eco-friendly than chemical conversion, but it is not yet economically competitive, mainly due the high cost of the needed enzymes. Development of enzymes with improved performances by enhancing their stability and specific activity is therefore pursued. Unlike dedicated energy crops and agro-industrial waste, the NW saccharification yield is generally low probably due to high lignin content, dense structure and the additional physical barrier constituted by toner’s ink and inorganic coating linked to the lignin (Chu and Feng 2013; Kim et al. 2006; Kuhad et al. 2010). In order to overcome this bottleneck, the evaluation of the most efficient pretreatment method and of a suitable tailor-made enzymatic mixture are the crucial steps.

This study is aimed at evaluating the feasibility of NW as feedstock for the fermentable sugars production by testing two different physicochemical pretreatment methods, ammonia fiber expansion (AFEX) and extractive ammonia fiber expansion (EA).

We have previously shown that tailor-made enzymatic cocktails including the endocellulase rCelStrep from *Streptomyces* sp. G12 recombinantly expressed in *Escherichia coli* (Amore et al. 2012b), the α-l-arabinofuranosidase rPoAbf from the fungus *Pleurotus ostreatus* recombinantly expressed in *Pichia pastoris* (Amore et al. 2012a) and/or its evolved variant rPoAbf F435Y/Y446F (Giacobbe et al. 2014) are suitable to improve the hydrolysis yields of AFEX pretreated corn stover and *Arundo donax* (Giacobbe et al. 2015). Based on these results, these enzymes were chosen in this study to investigate their ability to improve the saccharification of AFEX- and EA-treated NW, with the aim of valorizing this lignocellulosic MSW fraction.

## Materials and methods

### Feedstock

NW was used as feedstock for the saccharification experiments. The NW was collected in the recycle station of the Office for International Student and Scholar (OISS) at Michigan State University (MSU), East Lansing, Michigan and it was mainly composed of free weekly newspaper distributed in Lansing area. After shredding into pieces of 1 cm in wide and 20 cm in length, the NW was milled with a 2 mm diameter sieve and stored under dry conditions at room temperature until use.

### Compositional analysis

The composition analyses of NW were performed by acid hydrolysis according to the laboratory analytical procedures (LAPs) developed by the National Renewable Energy Laboratory (NREL) (Sluiter et al. 2010; Templeton et al. 2010):

- “Preparation of samples for compositional analysis” (Hames et al. 2008),
- “Determination of structural carbohydrates and lignin in biomass” (Sluiter et al. 2008c),
- “Determination of total solids in biomass and total dissolved solids in liquid process samples” (Sluiter et al. 2008a),
- “Determination of ash in biomass” (Sluiter et al. 2008b).

A moisture analyser was used to evaluate the moisture content. The acid insoluble lignin (Klason lignin) was detected by weighting the dried residue after total removal of the sugars. Monomeric sugars were quantified using a Biorad Aminex HPX-87H high-performance liquid chromatography (HPLC) column using 5 mM sulphuric acid as mobile phase.

### AFEX pretreatment

NW was subjected to AFEX pretreatment by varying reaction temperature (65–75 °C), moisture (10.7–25 % on dry weight basis), ammonia to biomass ratio (2.0:1 and 2.8:1) at fixed residence time of 15 min. AFEX was done in a high pressure stainless steel Parr reactor. Biomass was sprayed with water to reach appropriate moisture content after taking into consideration the moisture content of original biomass. Then the reactor was closed and vacuum applied to remove residual air in the reactor. The required amount of liquid ammonia was loaded using a ammonia delivery pump into a reactor pre-heated by external heating mantle. The biomass was mixed during AFEX pretreatment process for 15 min. As the temperature of reactor was increased, the pressure in the vessel increased (between 200 and 400 psi) relying on the ammonia to biomass loading. After the completion of pretreatment process, the pressure was released from the vessel and ammonia was vented in the hood. Subsequently, the biomass was transferred to a tray and dried in the hood overnight to remove residual ammonia. The dry pretreated biomass was stored in a sealed polythene bag at 4 °C until use.

### Extractive ammonia (EA) pretreatment

NW was subjected to EA pretreatment (reaction temperature: 120 °C; ammonia to biomass ratio: 3:1 kg/kg dry biomass; moisture content: 10 % on dry weight basis; fixed residence time: 15 min). EA was done in a high pressure stainless steel Parr reactor. Biomass was first sprayed with water to reach appropriate moisture content after taking into consideration the moisture content of original biomass. The required amount of liquid ammonia was loaded using a ammonia delivery pump into a reactor pre-heated by external heating mantle. The biomass was mixed during EA pretreatment process for 15 min. As the temperature of reactor was increased, the pressure in the vessel raised (between 600 and 800 psi) depending on the ammonia to biomass loading. After the completion of pretreatment process, extractives (mostly lignin) generated during the process along with liquid ammonia was collected via a sintered frit into another high pressure reactor. The collection pressure was released from the vessel and ammonia was vented in the hood to recover the extractives. The pretreated biomass was transferred to a tray and dried in the hood overnight to remove residual ammonia present in the biomass. Then dry EA treated biomass was stored in a sealed polythene bag in a refrigerator until further use.

### Commercial and purified enzymes and their sources

The commercially available complex of cellulases and hemicellulases Cellic^®^ CTec3 and the enzyme solution Cellic^®^ HTec3 from Novozymes (Denmark) were used in this study. Moreover, mixes of the following purified enzymes were tested. The four core fungal cellulases were cellobiohydrolase I (CBH I; glycoside hydrolase—GH—family 7A), cellobiohydrolase II (CBH II; GH family 6A), endoglucanase I (EG I; GH family 7B) and β-glucosidase (βG; GH family 3). CBH I, CBH II and EG I were purified from Spezyme CP (Danisco US Inc., Genencor Division, Rochester, NY) using four different chromatography methods: size exclusion, anion and cation exchange, hydrophobic interaction and affinity (Gao et al. 2010a, b); βG was purified from Novo 188 (Novozyme, Davis, CA, USA), by using anion and cation exchange chromatography (Gao et al. 2010a, b). The bacterial hemicellulases added to the core cellulases were xylanases (LX3, GH family 10; LX4, GH family 11), β-xylosidase (LβX; GH family 52) and α-arabinofuranosidase (LArb, GH family 51). LX3 and LX4 from *Clostridium thermocellum*, LβX from *Geobacillus stearothermophilus* and LArb from *Geobacillus* sp. G11MC16 were recombinantly expressed in *E. coli* BL21 (DE3) and purified using HIS-select nickel affinity chromatography (Gao et al. 2010a, b, 2011). The endoglucanase rCelStrep (GH family 12) was from *Streptomyces* sp. G12 and recombinantly expressed in *E. coli* (Amore et al. 2012b); the α-l-arabinofuranosidases rPoAbf wild type (GH family 51) from *P. ostreatus* and its mutant rPoAbf F435Y/Y446F were recombinantly expressed in *P. pastoris* (Amore et al. 2012a). These enzymes were purified by ammonium sulphate precipitation followed by hydrophobic interaction chromatography (Amore et al. 2012a, b).

### Enzymatic hydrolysis

The enzymatic hydrolyses were carried in 5 mL vials. An amount of pretreated biomass was added as to load 1 % (w/w) glucan in 2 mL total volume with the desired enzymes. The buffer solution was 50 mM citrate, pH 4.8. Microbial and fungal contaminations were prevented by adding sodium azide 0.5 mM. The hydrolysis parameters were: 50 °C, 250 rpm, 72 h. Sampling was collected every 24 h to evaluate carbohydrates hydrolysis.

Fifteen milligram per gram of glucan of the commercial enzymatic preparation Novozymes Cellic^®^ (60 % CTec3 and 40 % HTec3) was used for hydrolysis experiment to select the best pretreatment conditions.

The following enzymes were used for the hydrolysis of AFEX and EA pretreated NW. The MIX A was prepared including: CBH I, CBH II and EG I (3.32 mg/g glucan each), βG (2 mg/g glucan), LX3 and LX4 (1.66 mg/g glucan each), LβX and LArb (0.6 mg/g glucan each).

In the mix B, the EG I was replaced by an equal amount of endoglucanase rCelStrep; in the mix C the LArb was replaced by an equal amount of the α-l-arabinofuranosidase rPoAbf; in the mix D the LArb was replaced by the same amount of the mutant rPoAbf F435Y/Y446F.

Moreover, rCelStrep, rPoAbf or rPoAbf F435Y/Y446F were added alternatively or in combination to the MIX 1 containing 15 mg/g glucan of commercial preparation mix (60 % Cellic^®^ CTec3 and 40 % Cellic^®^ HTec3 from Novozymes). In particular the following enzymes were added: in the MIX 2, the endoglucanase rCelStrep (3.32 mg/g glucan); in the MIX 3 the α-l-arabinofuranosidase rPoAbf (0.6 mg/g glucan); in the MIX 4 the mutant rPoAbf F435Y/Y446F (0.6 mg/g glucan); in the MIX 5 both the endoglucanase rCelStrep (3.32 mg/g glucan) and the α-l-arabinofuranosidase rPoAbf (0.6 mg/g glucan); in the MIX 6 both the endoglucanase rCelStrep (3.32 mg/g glucan) and the mutant α-l-arabinofuranosidase rPoAbf F435Y/Y446F (0.6 mg/g glucan).

### Sugar analysis

Monomeric sugars concentration were determined by high performance liquid chromatography (HPLC). All experiments were performed in triplicate.

About 200 μl hydrolysate were collected in a centrifuge tube, heated to 100 °C for 10 min (to inactivate the enzymes), then spun down at 8000 rpm for 10 min and the supernatant was stored in a HPLC vial at −20 °C until further use. Monomeric sugars concentration in the hydrolysate was determined by HPLC using Biorad Aminex HPX-87P. Shimadzu HPLC Prominence system (Columbia, MD, USA) with a refractive index detector (RID) were used for analyzing the sugars. Water was used as the mobile phase at a fixed flow rate of 0.6 ml/min, with isocratic elution. The column temperature was maintained at 60 °C and the HPLC sample injection volume was 20 μl. Standard curves were generated using different concentrations of mixed sugars. A guard column with similar packing was used throughout the chromatography experiments.

The sugars conversion is calculated according to the following equations:

$${\text{Glucan conversion }}\;\left( \% \right) = \frac{{{\text{Glucose concentration in hydrolysate}} \;\left( {{{\text{g}} \mathord{\left/ {\vphantom {{\text{g L}}}} \right. \kern-0pt} {\text{L}}}} \right)}}{{{\text{Glucan concentration in loaded biomass}}\; \left( {{{\text{g}} \mathord{\left/ {\vphantom {{\text{g L}}}} \right. \kern-0pt} {\text{L}}}} \right) }} \times \frac{1}{1.11}$$

$${\text{Xylan conversion }} \left( \% \right) = \frac{{{\text{Xylose concentration in hydrolysate }}\;\left( {{{\text{g}} \mathord{\left/ {\vphantom {{\text{g L}}}} \right. \kern-0pt} {\text{L}}}} \right)}}{{{\text{Xylan concentration in loaded biomass }}\;\left( {{{\text{g}} \mathord{\left/ {\vphantom {{\text{g L}}}} \right. \kern-0pt} {\text{L}}}} \right) }} \times \frac{1}{1.136}$$

$${\text{Arabinan conversion }} \;\left( \% \right) = \frac{{{\text{Arabinose concentration in hydrolysate}} \;\left( {{{\text{g}} \mathord{\left/ {\vphantom {{\text{g L}}}} \right. \kern-0pt} {\text{L}}}} \right)}}{{{\text{Arabinan concentration in loaded biomass}} \;\left( {{{\text{g}} \mathord{\left/ {\vphantom {{\text{g L}}}} \right. \kern-0pt} {\text{L}}}} \right) }} \times \frac{1}{1.136}$$

$${\text{Mannan conversion }} \left( \% \right) = \frac{{{\text{Mannose concentration in hydrolysate}} \;\left( {{{\text{g}} \mathord{\left/ {\vphantom {{\text{g L}}}} \right. \kern-0pt} {\text{L}}}} \right)}}{{{\text{Mannan concentration in loaded biomass}} \;\left( {{{\text{g}} \mathord{\left/ {\vphantom {{\text{g L}}}} \right. \kern-0pt} {\text{L}}}} \right) }} \times \frac{1}{1.11}$$

where 1.11 is the ratio of MW_glucose (or mannose)_ (180.16 g/mol) to MW_glucan (or mannan)_ (162.14 g/mol) and 1.11 is the ratio of MW_xylose (or arabinose)_ (150.13 g/mol) to MW_xylan (or arabinan)_ (132.13 g/mol).

## Results

### Composition analysis and pretreatment of newspaper waste

Composition analysis of untreated NW was carried out and the results reported in Table 1 revealed a glucan content of ~44 %, a hemicellulose content of ~15 % and a Klason lignin content of ~25 %.

**Table 1:** Macromolecular composition of newspaper waste before and after EA pretreatment

	Newspaper waste	
	Untreated	EA pretreated
Moisture content	7.00 ± 1.00	9.75 ± 0.07
Ash	7.04 ± 0.14	7.25 ± 0.15
Structural carbohydrate		
Glucan	44.21 ± 4.02	41.36 ± 0.01
Xylan	5.11 ± 0.19	5.20 ± 0.22
Galactan	1.81 ± 0.13	1.84 ± 0.03
Arabinan	1.09 ± 0.09	1.73 ± 0.03
Mannan	9.83 ± 0.47	8.16 ± 0.05
Lignin		
Acid insoluble lignin	25.88 ± 2.48	30.03 ± 0.89
Acid soluble lignin	0.96 ± 0.01	1.05 ± 0.19
Composition closure	95.92	96.63

The macromolecular composition of AFEX pretreated NW is assumed to have the same composition of untreated NW (Table 1), due to the fact that, as previously reported, the AFEX pretreatement preserves the macrostructure of the lignocellulosic biomasses, reducing the degree of polymerization of (hemi)cellulose minimizing the degradation of the original carbohydrates (Holtzapple et al. 1991; Kumar et al. 2009). In order to select the best AFEX conditions to be used during the further saccharification experiments, three different sets of conditions were tested on NW (Table 2), varying ammonia loading, temperature and moisture content. These conditions were chosen based on the previous published screening of AFEX pretreatments on NW (Holtzapple et al. 1991, 1992).

**Table 2:** AFEX conditions tested on newspaper waste

AFEX conditions	Ammonia loading (kg:kg dry biomass)	Reaction temperature (°C)	Moisture content (%)	Fixed residence time (min)	Sugars conversion (after 72 h hydrolysis)	
					(%)	(g/L)
N1	2.8:1	65	10.7	15	29.58	4.62
N2	2.8:1	75	25		29.02	4.53
N3	2.0:1	75	25		26.14	4.08

**Fig. 1:**
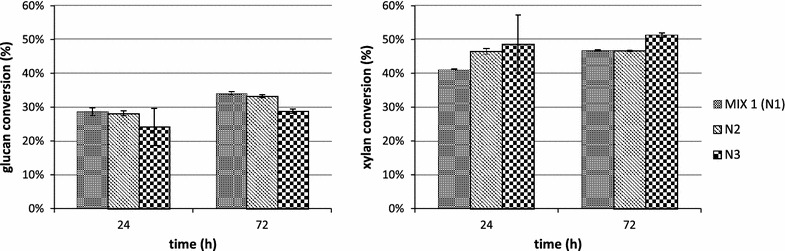
Glucan and xylan conversion during the hydrolysis of newspaper waste after three different AFEX pretreatment conditions: N1 (ammonia loading 2.8:1; 65 °C; 10.7 % moisture content; 15 min), N2 (ammonia loading 2.8:1; 75 °C; 25 % moisture content; 15 min) and N3 (ammonia loading 2.0:1; 65 °C; 10.7 % moisture content; 15 min)

The AFEX pretreated NW was subjected to enzymatic hydrolysis using the mix composed of 60 % Cellic^®^ CTec3 and 40 % Cellic^®^ HTec3 from Novozymes. As shown in Fig. 1, the maximum glucose and xylose yields were achieved after 72 h of hydrolysis with the AFEX pretreatment condition N1 (2.8 kg ammonia/kg dry biomass, 65 °C, and 10.7 % of moisture content) and N2 (2.8 kg ammonia/kg dry biomass, 75 °C, and 25 % of moisture content) and the maximum total sugars conversion was respectively 29.58 ± 0.35 and 29.02 ± 0.35 % (Table 2). The condition N1 was selected for the further hydrolysis experiments due to the milder reaction temperature of pretreatment (10 °C less than condition N2).

As an alternative pretreatment method, the EA was also tested on NW. The EA pretreatment converts native cellulose I to cellulose III, delignifying the biomass simultaneously (da Costa et al. 2015). Composition analysis of EA pretreated NW was carried out and the results showed that the NW still contains significant percentages of structural sugars after EA pretreatment (~58 %). In particular, the percentages of glucan content is ~41 %, the percentage of mannan content is ~8 % and the percentage of xylan content is ~5 %; the total lignin content is ~31 % (Table 1).

### Hydrolysis of newspaper waste by using the enzymes rCelStrep, rPoAbf and its variant in comparison with the enzymes EGI or LArb

As shown in several papers, a mix of cellulases and accessory hemicellulases is crucial to enhance hydrolysis of pretreated lignocellulosic biomasses (Gao et al. 2010a, b; Jørgensen et al. 2007). The MIX A, previously optimized for corn stover saccharification after AFEX pretreatment (Gao et al. 2011), was chosen as reference mix. This mixture contains the cellobiohydrolases CBHI and CBHII, the endo-glucanase EGI, the beta-glucosidase βG, the xylanases LX3 and LX4, the beta-xylosidase LβX and the α-l-arabinofuranosidase LArb. In order to evaluate the hydrolysis yield by using the enzymes rCelStrep, rPoAbf and rPoAbf F435Y/Y446F, these enzymes were replaced to EGI (MIX B) and to LArb (MIX C and MIX D), respectively.

**Fig. 2:**
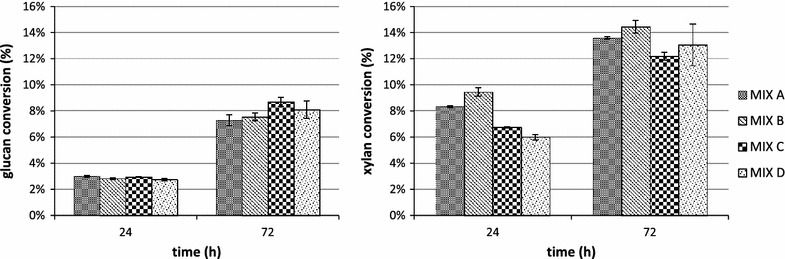
Glucan and xylan conversion during the hydrolysis of AFEX pretreated newspaper waste by using MIX A: CBH I, CBH II and EG I (3.32 mg/g glucan each), βG (2 mg/g glucan), LX3 and LX4 (1.66 mg/g glucan each), LβX and LArb (0.6 mg/g glucan each); MIX B (replacing EG I with endoglucanase rCelStrep); MIX C (replacing LArb with the α-l-arabinofuranosidase rPoAbf); MIX D (replacing LArb with mutant rPoAbf F435Y/Y446F)

**Fig. 3:**
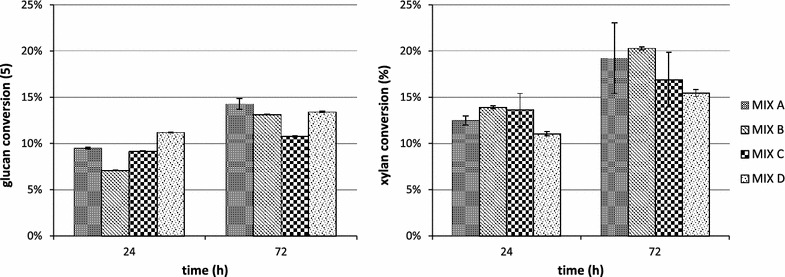
Glucan and xylan conversion during the hydrolysis of EA pretreated newspaper waste by using MIX A: CBH I, CBH II and EG I (3.32 mg/g glucan each), βG (2 mg/g glucan), LX3 and LX4 (1.66 mg/g glucan each), LβX and LArb (0.6 mg/g glucan each); MIX B (replacing EG I with endoglucanase rCelStrep); MIX C (replacing LArb with the α-l-arabinofuranosidase rPoAbf); MIX D (replacing LArb with mutant rPoAbf F435Y/Y446F)

The highest glucan, xylan, mannose and arabinose conversions were reached after 72 h of hydrolysis for all the tested biomasses, pretreatment methods and enzyme mixes. In Figs. 2 and 3 the conversion data for the most abundant polysaccharides (glucan and xylan) are reported.

For AFEX pretreated NW, as shown in Fig. 2, the glucan conversion reached 7.3 % for MIX A and did not increase significantly by using the other mixes. The xylan conversion was 13.59 % for MIX A and increased up to 14.44 % only for MIX B (corresponding to the substitution of rCelStrep to EGI), decreasing for the other mixes (12.2 % for MIX C and 13.1 % for MIX D). The arabinose conversion reached 7.61 % for MIX A, increased up to 8.86 % for MIX B and decreased to 4.4 % for MIX C and to 5.3 % for MIX D. The mannose conversion was undetectable for all mixes. In conclusion, there was no substantial improvement by replacing rCelStrep, rPoAbf or its variant to the corresponding enzymatic activities in the reference mix.

As shown in Fig. 3, for EA pretreated NW, the glucan conversion reached 14.3 % for MIX A and decreased for other three mixes (up to 13.1 % for MIX B, 10.7 % for MIX C and 13.4 % for MIX D). The xylan conversion was 19.2 % for MIX A and increased only for MIX B (up to 20.3 %), decreasing for MIX C (16.9 %) and for MIX D (15.4 %). The arabinose conversion reached 12.5 % for MIX A and decreased to 11.1 % for MIX B, 9.9 % for MIX C and 8.70 % for MIX D. The mannose conversion was less than 1 % for all the mixes. In conclusion, the maximum polysaccharides conversion yield (12.4 %) was obtained for MIX A.

### Evaluation of synergism between commercial enzyme preparation and rCelstrep and/or rPoAbf and its mutant

The enzymes rCelStrep, rPoAbf or its evolved variant rPoAbf F435Y/Y446F were added alternatively or in combination to 15 mg/g glucan of MIX 1 (60 % Cellic^®^ CTec3 and 40 % Cellic^®^ HTec3 from Novozymes) for the hydrolysis reactions of pretreated NW. The amount of enzyme loading was chosen based on the data obtained by Gao et al. (2011) and due to the promising results previously achieved for the hydrolysis of AFEX pretreated *Corn stover* and *A. donax* (Giacobbe et al. 2015).

**Fig. 4:**
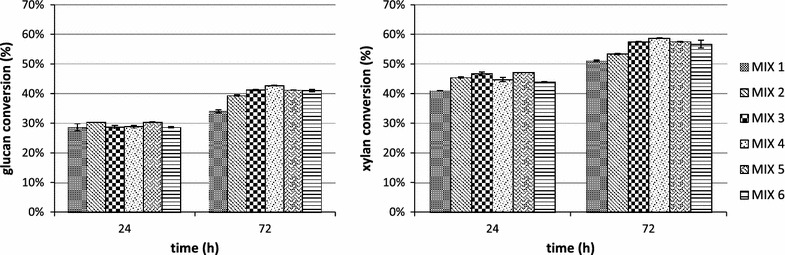
Glucan and xylan conversion during the hydrolysis of AFEX pretreated newspaper waste by using MIX 1 (15 mg/g of glucan of commercial enzymatic preparation Novozymes Cellic^®^—60 % CTec3 and 40 % HTec3; MIX 2 (adding 3.32 mg/g glucan of endoglucanase rCelStrep to MIX 1); MIX 3 (adding 0.6 mg/g glucan of the α-l-arabinofuranosidase rPoAbf to MIX 1); MIX 4 (adding 0.6 mg/g glucan of the mutant rPoAbf F435Y/Y446F to MIX 1); MIX 5 (adding 3.32 mg/g glucan of endoglucanase rCelStrep and 0.6 mg/g glucan of the α-l-arabinofuranosidase rPoAbf to MIX 1); MIX 6 (adding 3.32 mg/g glucan of endoglucanase rCelStrep and 0.6 mg/g glucan of the mutant rPoAbf F435Y/Y446F to MIX 1)

**Fig. 5:**
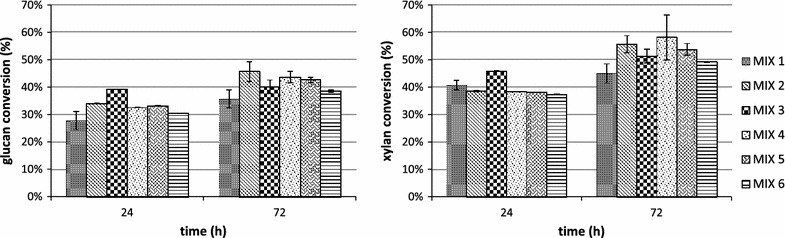
Glucan and xylan conversion during the hydrolysis of EA pretreated newspaper waste by using MIX 1 (15 mg/g of glucan of commercial enzymatic preparation Novozymes Cellic^®^—60 % CTec3 and 40 % HTec3; MIX 2 (adding 3.32 mg/g glucan of endoglucanase rCelStrep to MIX 1); MIX 3 (adding 0.6 mg/g glucan of the α-l-arabinofuranosidase rPoAbf to MIX 1); MIX 4 (adding 0.6 mg/g glucan of the mutant rPoAbf F435Y/Y446F to MIX A’); MIX 5 (adding 3.32 mg/g glucan of endoglucanase rCelStrep and 0.6 mg/g glucan of the α-l-arabinofuranosidase rPoAbf to MIX 1); MIX 6 (adding 3.32 mg/g glucan of endoglucanase rCelStrep and 0.6 mg/g glucan of the mutant rPoAbf F435Y/Y446F to MIX 1)

The highest glucan, xylan and arabinose conversion were reached after 72 h of hydrolysis for all tested biomasses, pretreatment methods and enzyme mixes. In Figs. 4 and 5 the conversion data for the most abundant polysaccharides (glucan and xylan) are reported.

As shown in Fig. 4, for AFEX pretreated NW, the glucan conversion reached 34.0 % for MIX 1 and increased up to 39.4 % for MIX 2, 41.2 % for MIX 3, 42.7 % for MIX 4, 41.22 % for MIX 5 and 41.0 % for MIX 6. The xylan conversion was 46.7 % for MIX 1 and increased up to 53.3 % for MIX 2, 57.5 % for MIX 3, 58.7 % for MIX 4, 57.50 % for MIX 5 and 56.7 % for MIX 6. The arabinose conversion reached 80.7 % for MIX 1 and decreased for all other mixes (67.3 % for MIX 2, 71.6 % for MIX 3, 70.8 % for MIX 4, 71. 7 % for MIX 5 and 70.0 % for MIX 6). The mannose conversion was undetectable for MIX 1 and reached 4.5 % for MIX 2, 6.1 % for MIX 3, 4.7 % for mix 4, 4.6 % for MIX 5 and 4.5 % for MIX 6. In conclusion, the maximum polysaccharides conversion (37.32 %) was obtained by adding rPoAbf F435Y/Y446F (0.6 mg/g glucan) to MIX 1.

As shown in Fig. 5, for the EA pretreated NW, the glucan conversion reached 35.6 % for MIX 1 and increased up to 45.6 % for MIX 2, 40.1 % for MIX 3, 43.6 % for MIX 4, 42.657 % for MIX 5 and 38.5 % for MIX 6. The xylan conversion was 44.9 % for MIX 1 and increased up to 55.6 % for MIX 2, 51.3 % for MIX 3, 58.1 % for MIX 4, 53.7 % for MIX 5 and 49.2 % for MIX 6. The arabinose conversion reached 49.0 % for MIX 1 and increased up to 52.1 % for MIX 2, 49.2 % for MIX 3, 53.7 % for MIX 4, 51.0 % for MIX 5 and 50.2 % for MIX 6. The mannose conversion reached 17.2 % for MIX 1 and decreased for all the other mixes (13.6 % for MIX 2, 12.8 % for MIX 3, 11.4 % for MIX 4, 13.0 % for MIX 5 and 10.0 % for MIX 6. In conclusion, the maximum polysaccharides conversion yield (40.80 %) was obtained for MIX 2, by adding rCelStrep (3.32 mg/g glucan) to MIX 1.

## Discussion

Composition analysis of untreated NW, assumed equal to the macromolecular composition of AFEX pretreated NW, as aforementioned, was in agreement with the values previously reported by Wang et al. (2012b, 2013), Subhedar et al. (2015), Sangkharak (2011) and Orozco et al. (2013) related to the untreated biomass. Furthermore, composition analysis of EA pretreated NW revealed that this technique, similarly to the AFEX pretreatment, does not significantly change the composition. Furthermore, the cellulose percentage after acid pretreatment, reported by Guerfali et al. (2015), and the holocellulose percentage, after alkaline pretreatment, reported by Wu et al. (2014), were comparable to data obtained after AFEX or EA method; contrariwise, the alpha cellulose and holocellulose percentage, reported by Sangkharak (2011), after pretreatment by using NaOH are both higher than those reported in this study, but the reaction conditions used for AFEX reaction are milder (65 versus 100 °C and residence time 15 min versus 4 h for AFEX and basic pretreatment respectively).

The hydrolysis of pretreated NW obtained by using mixes of purified enzymes showed that the EA method improve the saccharification in comparison with the AFEX, although the hydrolysis yields remained low in comparison to the data previously reported in literature. The maximum total polysaccharides conversion yield (for EA-pretreated-NW hydrolyzed by enzymatic MIX A) was similar to the yields reported by Ali and Khan Mohd (2011) after basic pretreatment and six days of microbial hydrolysis by *Aspergillus niger* and lower than the most recent reported results (Wua et al. 2014; Chu and Feng 2013; Wang et al. 2012b; Guerfali et al. 2015; Subhedar and Gogate 2014; Kim et al. 2006; Xin et al. 2010). Moreover, the glucan conversion yield was very low (not exceeding 7.3 %). This is the major drawback as glucose is the main carbon source for a wide range of industrial fermentation processes by using well-known microorganisms capable of using mainly hexoses (Jang et al. 2012). These results showed that the enzymatic mix previously optimized for pretreated corn stover saccharification is not suitable for the hydrolysis of NW after AFEX or EA pretreatment. Moreover, the replacement of the enzymes rCelStrep, rPoAbf and rPoAbf F435Y/Y446F respectively to EGI and LArb did not change significantly the saccharification yield. These data suggested that the purified enzymes are not probably able to overcome the physical barrier constituted by toner’s ink and inorganic coating that remain linked to the lignin after pretreatment.

On the other hand, the AFEX and EA pretreatments had comparable effects on the polysaccharides conversion yield after the hydrolysis of NW by using Cellic^®^ CTec3 and HTec3 and the addition of rCelStrep, rPoAbf or its evolved variant rPoAbf F435Y/Y446F to the commercial mix improved the sacharification process. In particular, the maximum glucan conversion yields reached for AFEX pretreated NW and EA pretreated NW were obtained by an additive effect of the α-l-arabinofuraosidase rPoAbf F435Y/Y446F and the endocellulase rCelStrep, respectively, in addition of the array of (hemi)cellulase activities present in the commercial mix. The increase of the glucan conversion yield was respectively of 25 and 28 % more than the yields obtained by hydrolysis with commercial enzymes without any addition. The best obtained sugars conversion yield was higher than or comparable to those reported in recent manuscripts adopting hydrolysis conditions similar to those used in this study. In particular, the maximum obtained glucan conversion yields were comparable to those described by Wang et al. (2012b) after enzymatic hydrolysis using 5 % (w/w) biomass and 64 mg/g glucan of commercial enzymes (~3 times more than those loaded in this study). Moreover, Wu et al. (2014) obtained sugar conversion yields comparable to this study after basic pretreatment and combining acid and enzymatic hydrolysis. Subhedar and Gogate (2014) obtained ~31 % of total sugars yield (lower than the results of this study) by using enzymatic hydrolysis and the yield increased only after an ultrasound-assisted enzymatic hydrolysis. However, the best obtained results in this study are lower than those reported by Guerfali et al. (2015) and Kim et al. (2006) that add the action of surfactant agents to the enzymatic hydrolysis.

In conclusion, the feasibility of NW pretreatment by both AFEX and EA were demonstrated. The results showed no substantial differences between the two tested methods on hydrolysis yield. However, the AFEX was the best pretreatment technique mainly due to the mild reaction conditions. Moreover, the best sugars conversion yield obtained by adding the recombinant enzymes to the commercial mixture was higher than or comparable to those reported in previous studies adopting similar hydrolysis conditions. These promising but not optimal results suggest that the process can be optimized in order to further enhance the NW hydrolysis yield.
